# Radiation therapy for atypical and anaplastic meningiomas: an overview of current results and controversial issues

**DOI:** 10.1007/s10143-022-01806-3

**Published:** 2022-06-04

**Authors:** Lorenzo Vagnoni, Sami Aburas, Martina Giraffa, Ivana Russo, Vito Chiarella, Sergio Paolini, Paolo Tini, Giuseppe Minniti

**Affiliations:** 1 Department of Medicine, Surgery and Neurosciences, University of Siena, 53100 Siena, Italy; 2 UPMC Hillman Cancer Center, San Pietro Hospital FBF, Rome, Italy; 3 UPMC Hillman Cancer Center, Villa Maria, Mirabella Eclano (AV), Italy; 4 IRCCS Neuromed, 86077 Pozzilli (IS), Italy

**Keywords:** Atypical meningioma, Anaplastic meningioma, Radiation therapy, Fractionated radiotherapy, Stereotactic radiosurgery

## Abstract

Meningiomas are the most common intracranial tumors. Most meningiomas are WHO grade 1 tumors whereas less than one-quarter of all meningiomas are classified as atypical (WHO grade 2) and anaplastic (WHO grade 3) tumors, based on local invasiveness and cellular features of atypia. Surgical resection remains the cornerstone of meningioma therapy and represents the definitive treatment for the majority of patients; however, grade 2 and grade 3 meningiomas display more aggressive behavior and are difficult to treat. Several retrospective series have shown the efficacy and safety of postoperative adjuvant external beam radiation therapy (RT) for patients with atypical and anaplastic meningiomas. More recently, two phase II prospective trials by the Radiation Therapy Oncology Group (RTOG 0539) and the European Organisation for Research and Treatment of Cancer (EORTC 2042) have confirmed the potential benefits of fractionated RT for patients with intermediate and high-risk meningiomas; however, several issues remain a matter of debate. Controversial topics include the timing of radiation treatment in patients with totally resected atypical meningiomas, the optimal radiation technique, dose and fractionation, and treatment planning/target delineation. Ongoing randomized trials are evaluating the efficacy of early adjuvant RT over observation in patients undergoing gross total resection.

## Introduction

Meningiomas are the most common primary intracranial tumors and account for more than one-third of all brain tumors [1]. Based on local invasiveness and cellular features of atypia, meningiomas are histologically characterized as benign (grade 1), atypical (grade 2), or anaplastic (grade 3) tumors by the latest World Health Organization (WHO) classification scheme [2]; accordingly, the proportion of meningiomas that have been classified as atypical and anaplastic meningiomas is around 20–25% and 1–3%, respectively [3]. For both, surgical resection is the first choice of treatment; however, a significant proportion of tumors display a more aggressive behavior associated with an approximately 6–8-fold increased risk of recurrence and a significantly higher risk of dying of tumor progression compared to benign meningiomas [4, 5].

Beyond surgery, external beam radiation therapy (RT) has been usually recommended to increase local control after resection of grade 2 and 3 tumors [6]. The evidence supporting this treatment recommendation largely comes from systematic reviews including retrospective series [7–9] and two recent nonrandomized observational prospective trials conducted by the Radiation Therapy Oncology Group (RTOG 0539) [4, 10] and the European Organisation for Research and Treatment of Cancer (EORTC 22042) [11]; however, several issues remains a matter of debate, including the timing of the treatment (early versus delayed postoperative RT), the optimal radiation technique, and types of radiation dose and fractionation. One of the most controversial topics is the superiority of early adjuvant RT over observation in reducing the risk of tumor recurrence after gross total surgical resection in patients with atypical meningiomas. In addition, there is concern regarding potential risks of RT-related toxicity, which include but are not limited to neurocognitive impairment, hypopituitarism, and the development of a second tumor. Hopefully, these important questions will be answered by two prospective controlled phase III trials where patients were randomized to receive adjuvant RT or observation after surgical resection of an atypical meningioma: the recently closed ROAM/EORTC 1308 trial [12] and the ongoing NRG-BN003 (ClinicalTrials.gov Identifier: NCT03180268) trial.

In this review, we discuss some of the most recent advances in radiation treatment of patients with atypical and anaplastic meningiomas, as well as evidence supporting its use in the context of different clinical situations. The safety and efficacy of different radiation approaches and techniques were also examined.

## Histopathologic classification

The systematic adoption of the histopathologic criteria provided by the 2016 update of the WHO classification of brain tumors has markedly increased the relative proportion of atypical and anaplastic meningiomas [13]. Both tumors exhibit a much greater recurrence rate compared to benign meningiomas, which negatively impacts survival. As confirmed by the latest WHO classification, tumors with low mitotic rate (less than 4 per 10 high power fields (HPF) are generally classified as benign, WHO grade 1 tumors. For grade 2 atypical meningiomas, brain invasion or a mitotic count of 4–19 per HPF are a sufficient criterion for the diagnosis [2]. Atypical meningiomas can also be diagnosed in presence of 3 or more of the following properties: sheetlike growth, spontaneous necrosis, high cellularity, prominent nucleoli, and small cells with high nuclear-cytoplasmic ratio. Grade 3 anaplastic meningiomas are characterized by elevated mitotic activity (20 or more per HPF) or frank anaplasia. In addition, specific histologic subtypes such as clear cell or chordoid cell meningiomas are classified as grade 2, and rhabdoid or papillary meningiomas as grade 3 tumors. A new finding of WHO 2021 classification is the inclusion of several molecular biomarkers associated with the classification and grading of meningiomas, e.g., *SMARCE1* in clear cell subtype, *BAP1* in rhabdoid and papillary subtypes, *KLF4/TRAF7* in secretory subtype mutations, and *TERT* promoter mutation and/or homozygous deletion of *CDKN2A/B* in anaplastic meningiomas. When these criteria are applied, up to 3 and to 25% of all meningiomas are atypical or anaplastic.

## Radiation techniques

Assuming that RT is of value in improving tumor control, new advanced radiation techniques can provide excellent target dose coverage, precise target localization, and accurate dose delivery [14]. For large postoperative resection cavity and/or residual tumors, sophisticated techniques using intensity-modulated radiotherapy (IMRT) or volumetric modulated arc therapy (VMAT) allow highly conformal dose distribution and should be preferred over three-dimensional (3D) conformal RT. Stereotactic radiation techniques, given as either radiosurgery (SRS) or hypofractionated radiotherapy (SRT), have been employed in patients with residual or recurrent atypical and anaplastic meningiomas [15–28]. The main advantage of stereotactic techniques is their ability to achieve a steep dose fall-off at the edge of the target volume lowering the radiation dose to surrounding brain structures, then limiting the potential toxicity of treatments. Current stereotactic techniques include Gamma Knife (Elekta Instruments AB, Stockholm, Sweden) and linear accelerator (LINAC)-based SRS systems, such as CyberKnife (Accuray, Sunnyvale, CA, USA) or Novalis (NTx) (BrainLAB AG, Feldkirchen, Germany). Patients receiving Gamma Knife SRS are traditionally placed in a rigid stereotactic frame with a submillimetric target accuracy while those treated with LINAC-based SRS systems are usually immobilized in a high precision frameless stereotactic mask fixation system. A submillimeter accuracy of patient positioning in the treatment room is achieved using modern image-guided radiation therapy (IGRT) technologies, such as orthogonal x-rays (ExacTrac®Xray 6D system) or cone-beam CT (CBCT) [29]. Although dosimetric characteristics of these SRS systems can be different, no comparative studies have demonstrated the clinical superiority of one technique over another in patients with brain tumors in terms of local control and treatment-related toxicity.

Protons have been employed for skull base tumors either as fractionated RT or as SRS [14]. A radiobiological advantage of protons over photons is that they deposit most of their energy at the end of their range, with very little exit dose beyond the target volume. This narrow region of energy deposition is known as the Bragg peak and it may allow for a lower integral dose delivered to the surrounding normal tissues with protons as compared with photons. Because of the limited number of published series and their retrospective nature (see chapter below), current clinical data do not allow any definitive conclusion about the superiority of proton-based over photon-based techniques in terms of effectiveness and long-term toxicity.

## Imaging and tumor delineation

For resected tumors, the treatment planning is based on postoperative MRI, although preoperative MRI may provide useful information on the initial extent of disease and persistent postoperative brain infiltration. The gross tumor volume (GTV) delineation is based on the resection cavity plus any residual tumor using pre- and post-contrast T1-weighted postoperative magnetic resonance imaging (MRI) sequences, without the inclusion of the perilesional edema [30]. Additional images that can help to improve target delineation include T2-weighted high-resolution gradient and fast spin-echo sequences with and without fat suppression, and fluid-attenuated inversion recovery (FLAIR) sequences which can help to assess the extent of peritumoral edema and dural tail abnormalities [11, 31]. In selected cases, PET imaging mainly with DOTATOC-tracers or DOTANOC-tracers has shown to improve target volume definition, e.g. patients with large tumors infiltrating the parapharyngeal soft tissues or for those located in the bony structures which are difficult to be distinguished on MRI and CT [32, 33]. The clinical target volume (CTV), defined as the volume of tissue that contains any microscopic disease and potential paths of microscopic spread, comprises the preoperative tumor bed and a geometrical expansion of 10 mm around the GTV, which may be reduced to 5 mm around anatomic barriers, such as non-infiltrated bone or non-infiltrated brain. The CTV can be extended along the dura up to 20 mm to encompass thickened dural tail or clearly involved hyperostotic bone, especially in the area of adjacent reactive dura. Depending upon the localization method and reproducibility, an institution-specific margin of 0.3–0.5 cm is usually added to the CTV to generate the planning target volume (PTV). For planning purposes, MRI scans are subsequently fused with thin-slice non-contrast-enhanced CT scans. Of note, CT scans may have a complementary role in the imaging of skull base, specifically showing the pattern of bone involvement, e.g. hyperostosis and osteolysis, as well identifying intratumoral calcification better than MRI [34].

## Radiation therapy outcome

### Fractionated RT following resection of atypical meningiomas

Fractionated RT remains an important component of the therapeutic armamentarium for the treatment of patients with atypical meningiomas [4, 5, 10, 35–61]. Selected studies reporting clinical outcomes of patients with atypical meningioma following surgery with or without adjuvant RT are summarized in Table 1 [4, 5, 10, 35, 36, 38–41, 43–46, 48–53, 56, 57, 59, 61].

**Table 1:** Summary of main published studies on radiotherapy for atypical (grade 2) meningiomas

Authors	Pts No	Treatment modality	Median dose Gy	Median fsollow-UP Months	Progression-free survival, %	Overall survival, %	Toxicity
Aghi et al. 2009 [2]	108	Surgery (*n*=100) Surgery+RT (n=8)	CRT 60.2	39	44 at 5 years 100 at 5 years	NA	12.5%
Mair et al. 2011 [54]	114	Surgery (*n* =84) Surgery+RT (*n*=30)	CRT 51.8	NA	40 at 5 years 60 at 5 years	NA	NA
Hardesty et al. 2013 [32]	228	Surgery (*n* =157) Surgery+RT (*n* =71)	SRS (*n*=32) IMRT 54 (*n*=39)	52	74 at 5 years (SRS) 60 at 5 years (IMRT)	NA	NA
Park et al. 2013 [62]	83	Surgery (*n* =56) Surgery+RT (*n* =27)	CRT 61.2	43	44.3 at 5 years 58.7 at 5 years	90 at 5 years 62 at 10 years	No severe
Aizer et al. 2014 [3]	91	Surgery (*n* =57) Surgery+RT (*n* =34)	CRT 60 SRS 16 (*n*=1)	57	67.8 at 5 years 82.6 at 5 years	NA	NA
Hammouche et al. 2014 [30]	79	Surgery (*n*=43) Surgery+RT (*n*=36)	CRT 56.2	50	56 at 5 years 51 at 5 years	81 at 5 years	NA
Yoon et al. 2015 [63]	158	Surgery (*n*=135) Surgery+RT (*n*=23)	CRT 57 SRS 14 (*n*=11)	32	88 59	83 at 5 years 89 at 5 years	NA
Jenkinson et al. 2016 [39]	133	Surgery (*n*=97) Surgery+RT (*n*=36)	CRT 60	57.4	75.8 at 5 years 71.9 at 5 years	72.7 at 5 years 67.8 at 5 years	NA
Dohm A et al. 2017 [21]	115	Surgery (*n*=52) Surgery+RT (*n*=63)	CRT 55.7 SRS 14.5 (*n*=16)	36.9	27 at 5 years 59 at 5 years	55 at 5 years 75 at 5 years	Grade 3 (*n*=7) Grade 4 ( *n*=1)
Champeaux C et al. 2017 [12]	215	Surgery (*n*=150) Surgery+RT (*n*=65)	CRT 54 (*n*= 56) SRS 16 (*n*= 9)	54	NA	NA	NA
Weber DH et al. 2018 (EORTC 22042–26042) [64]	78*	Surgery (*n*=22) Surgery+RT (*n*=56)	CRT 60 Gy	61	88.7 at 3 years (Surgery+RT)	98.2 at 3 years (Surgery+RT)	Grade 3 or 4, 14.3%
Rogers L et al. 2018 (RTOG 0539) [65]	56	Surgery (*n*=4) Surgery+RT (*n*=52)	IMRT 54 (*n*= 44) CRT 54 (*n*= 8)	44	93.8 at 3 years	96 at 3 years	No grade 3 toxicity
Masalha W et al. 2018 [56]	161	Surgery (*n*=128) Surgery+RT (*n*=33)	CRT 55-57	NA	76, 64,and 57 at 3,5,and 10 years	NA	NA
Chen WC et al. 2018 [13]	182	Surgery (*n*=140) Surgery+RT (*n*=42)	CRT 59.4 (*n*=36) SRS (*n*=6)	52	82 at 5 years	85 at 5 years	Grade 2 (*n*=5)
Budohoski KP et al. 2018 [9]	220	Surgery (*n*= 205) Surgery+RT (*n*=57)	*N*A	24	59 and 19 at 5 and 10 years	87 and 69 at 5 and 10 years	NA
Zhi M et al. 2019 [66]	149	Surgery (*n*=96) Surgery+RT (*n*=53)	CRT 59.4	74.2	76 and 58 at 5 and 10 years	86 and 68 at at 5 and 10 years	NA
Li H et al. 2019 [49]	302	Surgery (*n*=227) Surgery+RT (*n*=75)	CRT 54**	41.6	47.7 at 5 years	78.8 at 5 years	NA
Wang F et al. 2019 [67]	263	Surgery (*n*= 177) Surgery+RT (*n*=86)	CRT 56	41	41	NA	NA
Zeng Q et al. 2019 [68]	1014	Surgery (*n*=727) Surgery+RT (*n*= 315)	*N*A	NA	NA	79 at 5 years	NA
Keric N et al. 2020 [42]	258	Surgery (*n*=212) Surgery+RT (*n*=46)	IMRT 56	31	70.7 at 5 years	NA	No grade 3 toxicity
Poulen G et al. 2020 [69]	88*	Surgery (*n*=69) Surgery+RT (*n*=19)	CRT 56	69.2	55.8 at 5 years	89.7 at 5 years	NA
Rogers L et al. 2020 (RTOG 0539) [65]	53	Surgery+RT (*n*=53)	IMRT 60 (*n*=52 ) CRT 60 (*n*=1 )	48	58.8 at 3 years	78.6 and 59.1 at 3 and 5 years	Limited to Grades 1-3. Grade5 (*n*=1)
Lee et al. 2021 [48]	230	Surgery (*n*=179) Surgery+RT (*n*=51)	FSRT 59.4 (*n*=48) SRS 12-15 (*n*=3)	81	79 and 64 at 5 and 10 years (Surgery+RT)	91 and 85 at 5 and 10 years (Surgery+RT)	NA

*RT*, radiation therapy; *CRT*, conventional radiation therapy; *SRS*, stereotactic radiosurgery; *IMRT*, intensity-modulated radiation therapy; *FSRT*, fractionated stereotactic radiotherapy; *series include both grade 2 (*n*=69) and grade 3 (*n*=9) meningiomas; ** four patients received SRS; *NA*, not assessed

Results of two prospective phase II trials have been recently published by the RTOG and the EORTC [4, 11, 53]*.* The first report of The NRG Oncology/RTOG 0539 trial reported the initial outcome for 48 patients with intermediate-risk meningiomas, i.e., recurrent WHO grade 1 or newly diagnosed WHO grade 2 tumors after gross total resection, who were treated with IMRT or 3D conformal RT using doses of 54 Gy given in 30 fractions [4]. The estimated 3-year progression-free survival, overall survival, and local failure rates were 93.8%, 96%, and 4.1%, respectively. Clinical outcomes were similar between patients with recurrent benign meningiomas and atypical meningiomas receiving gross total resection. Adverse events were limited to grade 1 and grade 2 only. In a second report from the same trial, Rogers et al. [10] reported the clinical outcome for 53 patients with a high-risk meningioma, defined by new or recurrent anaplastic or recurrent atypical meningioma of any resection extent, or new atypical tumor after subtotal resection; treatment consisted of IMRT using simultaneous integrated boost, with the higher-dose volume receiving 60 Gy and lower-dose volume receiving 54 Gy in the same 30 fractions.

At a median follow-up of four years, 3-year progression-free survival was 58.8%, local control 68.9%, and overall survival 78.6%. Combined acute and late adverse events occurred in about 40% of patients and were limited to grades 1 to 3, except for a single necrosis-related grade 5 event. In the EORTC 22042-26042 phase II study, fifty-six patients with newly diagnosed WHO grade 2 meningioma who underwent gross total resection received adjuvant fractionated RT with a dose of 60 Gy delivered in 2 Gy per fraction [57]. Five patients did not receive the planned radiation dose: three patients prematurely stopped RT due to grade 3 cerebrospinal fluid leakage (unrelated to RT), vomiting, and epidermitis on scar, and two patients received 70 Gy instead of the planned 60 Gy**.** The estimated 3-year progression-free survival, overall survival, and local failure were 88.7%, 98.2%, and 14.3%, respectively, with a late toxicity of grade 3 or more observed in about 14% of patients.

The effectiveness of postoperative adjuvant RT in patients with atypical meningiomas has been evaluated in several retrospective series [5, 35, 36, 38–41, 43–46, 48–53, 56, 59, 61] (Table 1). A recent meta-analysis of 17 studies published between January 200 and January 2019 and including 2008 patients who have undergone gross total resection of atypical meningiomas showed a significant improvement in 5-year local control and progression-free survival rates for those receiving adjuvant RT [9]. Local control, progression-free survival, and overall survival rates were 82.2%, 84.1%, and 79%, respectively, for patients treated with adjuvant RT, and 71%, 71.9%, and 81.5%, respectively, for those not receiving the treatment. Lee et al. [22] reported the outcome of 179 patients who underwent surveillance versus 51 patients who received postoperative adjuvant RT with photons (39%) or protons (57%) after resection of an atypical meningioma. Compared with patients who underwent surveillance, patients who received adjuvant RT showed better progression-free survival; 5-year and 10-year rates were 79% and 64%, respectively, in the adjuvant RT group, versus 62% and 54%, respectively, in the surveillance group (log-rank *p*=0.03). Rates were significantly better in the adjuvant RT group after either gross total resection or subtotal resection; however, analysis of overall survival showed no difference between groups. Five-year and 10-year overall survival rates were 91% and 85%, respectively, in the adjuvant RT group, and 94% and 88%, respectively, in the surveillance group. In another series of 91 patients with atypical meningioma who received or who did not receive adjuvant RT at Dana-Farber/Brigham and Women’s Cancer Center between 1997 and 2011, Aizer et al. [36] observed 5-year local control rates of 82.6% and 67.8% in patients who did and did not receive RT, respectively (*p*=0.04). In multivariate analysis, the association between RT and local recurrence was significant (HR, 0.24; 95% CI, 0.06–0.91; *p*=0.04); however, no differences in overall survival were seen between groups. In another series of 108 patients with an atypical meningioma who underwent gross total resection at the University of California from 1993 to 2004, Aghi et al. [35] observed actuarial tumor recurrence rates of 41% at 5 years and 48% at 10 years. Adjuvant RT was associated with a trend toward decreased local recurrence (*p*=0.1) in eight patients who underwent gross total resection. Better progression-free survival rates in patients receiving postoperative RT compared with those who did not have been observed in few other retrospective studies [35, 36, 41, 50, 52, 55].

In contrast, some other studies showed no significant advantages in terms of either overall survival or progression-free survival for patients undergoing adjuvant RT [45, 59, 62, 70]. In a series of 158 patients with atypical meningiomas treated at the University of Wisconsin between 2000 and 2010, Yoon et al. [59] did not observe any beneficial impact of adjuvant RT on disease-free survival, irrespective of the extent of resection; survival rates were 89% for patients receiving gross total resection and 83% for those having subtotal resection. In another retrospective series of 133 patients treated between 2001 and 2010 in 3 different UK centres, Jenkinson et al. [45] reported similar outcomes for patients who received surgery with or without postoperative RT. Following gross total resection, 5-year overall survival and progression-free survival rates were 77.0% and 82%, respectively, in patients who received early adjuvant RT, and 75.7% and 79.3%, respectively, in patients who did not receive adjuvant treatment. Stessin et al*.* [70] published a Surveillance, Epidemiology, and End-Results (SEER)-based analysis of 657 patients who were diagnosed with atypical and anaplastic meningiomas in the period 1988–2007. Amongst a total of 244 who received adjuvant RT, the treatment was not associated with survival benefit even after stratification by grade, the extent of resection, size and anatomical location of the tumor, year of diagnosis, race, age, and sex. In addition, analysis of cases diagnosed after the WHO 2000 reclassification of meningiomas showed that RT led to inferior overall survival. Using the National Cancer Database, Wang et al*.* [62] have recently compared the survival outcome in 2515 patients with atypical meningioma diagnosed according to the 2007 WHO classification, treated with or without early postoperative RT after surgical resection. Gross total resection was associated with improved overall survival compared to subtotal resection; however, the favorable impact of adjuvant RT on survival was only seen in patients who underwent subtotal resection.

Overall, most studies indicate that adjuvant RT improves progression-free survival in patients with atypical meningiomas. The rate of tumor progression following subtotal resection is higher than that seen following gross total resection; however, the superiority of adjuvant RT over observation for totally excised atypical meningiomas in terms of overall survival remains a controversial issue. Although several studies showed a trend toward clinical benefit with adjuvant RT after gross total resection, the small number of patients evaluated, different WHO criteria for defining atypical meningiomas over the last decades, and the retrospective nature of published studies preclude any meaningful conclusion on whether adjuvant RT improves outcomes over nonirradiated patients. In this regard, the ongoing phase III randomized NRG-BN-003 trial and the recently closed ROAM/EORTC 1308 trial comparing surgery plus adjuvant RT with surgery alone in grade 2 meningioma following gross total resection will help answer the important clinical question on the efficacy of early postoperative RT. The primary outcome measure is progression-free survival (i.e., time to MRI evidence of tumor recurrence) and secondary outcome measures include radiation treatment-related toxicity, the quality of life, neurocognitive function, time to second-line treatment, and overall survival. Importantly, secondary analysis of trials will help to identify molecular features that will predict most benefit for patients receiving adjuvant RT. The results of this potentially practice-changing trial will be available in 2025.

### Fractionated RT following resection of anaplastic meningiomas

Few retrospective studies have evaluated the efficacy of RT in patients with anaplastic meningiomas [65, 69, 71–79]. A summary of selected published series is shown in Table 2 [57, 58, 69, 71–73, 75–77, 79]. Using total radiation doses of 54–60 Gy delivered in 1.8–2 Gy per fraction, the reported median 5-year progression-free survival and survial rates range from 29 to 80%, and from 27 to 81%, respectively. In a series of 7811 patients with WHO grade 2 and 1936 patients with grade 3 meningiomas obtained from the U.S. National Cancer Database who underwent surgical resection and RT from 2004 to 2014, alone or in combination, 5-year overall survival rates were 75.9% and 55.4%, respectively (*p* <.0001) [55].

**Table 2:** Summary of main published studies on radiotherapy for anaplastic (grade 3) meningiomas

Authors	Pts No	Treatment modality	Median dose Gy	Median follow-up months	Progression-free survival, %	Overall survival, %	Toxicity
Dziuk et al. 1998 [22]	38	Surgery (*n*=19) Surgery+RT (*n*=19)	54	3 to 144	74 and 25 at 2 and 5 years	NA	NA
Yang et al. 2008 [80]	24	Surgery (*n*=7) Surgery+RT (*n*=17)	SRS 13	42.5	50 and 29 at 3 and 5 years	55 and 35 at 3 and 5 years	NA
Rosenberg et al. 2009 [75]	13	Surgery (*n*=4) Surgery+RT (*n*=9)	CRT50–60 SRS 14–24	NA	52, 17, and 8.7 at 1,2 and 3 years	47 and 12.2 at 5 and 8 years	Necrosis (*n*=2)
Sughrue et al. 2010 [81]	63	Surgery +RT (*n*=63)	NA	60	80, 57, and 40 at 2, 5 and 10 years	82, 61, and 40 at 2, 5 and 10 years	19% neurological morbidity
Sumner et al. 2017 [82]	190	Surgery (*n*=101) Surgery+RT (*n*=89)	NA	56.4	NA	94.7 (surgery+RT) and 76.1 (RT) at 2 years	NA
Champeaux et al. 2019 [12]	178*	Surgery+RT (*n*=53)	59.4	54	NA	77.7, 40, and 27.9 at 1, 5 and 10 years	NA
Rogers et al. 2019 (RTOG 0539) [65]	57**	Surgery+RT (*n*=120)	IMRT 60 (*n*=52) CRT 60 (*n*=1)	48	59.2 at 3 years	78.6 at 3 years	Majority of patients had grade 1–3 toxicity. Grade 5 (*n*=1).
Masalha et al. 2019 [55]	36	Surgery (*n*=8) Surgery+RT (*n*=21)	FSRT59.4 (*n*=21)	49	52 and 19 at 1 and 3 years (Surgey+RT)	71 and 19 at 3 and 5 years	NA
Zhou H et al. 2019 [83]	254	Surgery (*n*=103) Surgery+RT (*n*=151)	CRT 56 (*n*=151)	34.5	NA	57.8 at 5 years (Surgery+RT)	NA
Alhourani et al. 2019 [4]	178	Surgery+RT (*n*=80)	IMRT 54 (*n*=73) SRS (*n*=7)	120	NA	32.8 months	NA

*RT*, radiation therapy; *CRT,* conventional radiation therapy; *SRS*, stereotactic radiosurgery; *IMRT*, intensity-modulated radiation therapy; *FSRT*, fractionated stereotactic radiotherapy; *67 patients had prior grade 1 or 2 meningiomas; ** series includes grade 3 meningiomas and new or recurrent grade 2 meningiomas; *NA*, not assessed

Champeaux et al. [72] reported a multicenter retrospective study of 178 patients treated between 1989 and 2017 for a anaplastic meningioma at six different international institutions. Median overall survival time and 5-year survival rates were 2.9 years and 27.9%, respectively; age <65 years, gross total resection, and adjuvant RT that emerged as independent prognostic factors for survival. Dziuk et al. [69] reported the outcome of 38 patients with an anaplastic meningioma who received (*n*=19) or did not receive (*n*=19) adjuvant RT. Adjuvant irradiation following gross total resection increased the 5-year progression-free survival rates from 15 to 80% (*p*=0.002). In contrast, recurrence rates after incomplete resection were similar between groups (100% vs 80%), with no survivors at 60 months. In another series of 24 patients with anaplastic meningiomas, Yang et al. [58] observed better overall survival and progression-free survival times in 17 patients who received adjuvant RT as compared with 7 patients who did not; however, the reported 5-year overall survival and progression-free survival rates were dismal in both groups, being 35% and 29%, respectively.

In contrast, other series failed to demonstrate a significant improvement in overall survival and progression-free survival times in patients receiving adjuvant RT [71, 79]. In a retrospective cohort of patients with atypical meningioma extracted from the National Cancer Database (NCDB) and diagnosed between 2004 and 2015, Alhourani et al. [71] evaluated the outcome of those patients with at least 10 years of follow-up after surgery and postoperative RT. The adjuvant treatment was associated with significantly improved local control; however, the median survival time was not significantly different (32.8 months for adjuvant RT vs. 38.5 months for no RT; *p* = 0.57, log-rank test).

In summary, anaplastic meningiomas are highly likely to recur regardless of resection status. In most of the retrospective published studies, adjuvant RT is associated with improved progression-free survival and overall survival; however, no prospective studies have compared surgery plus adjuvant RT versus surgery alone and definitive conclusions on the superiority of RT over observation cannot be drawn. Regarding the radiation techniques, fractionated RT given as adjuvant treatment is the most used type of irradiation, whereas SRS is usually reserved for small-to-moderate recurrent tumors.

### Radiosurgery

Adjuvant treatment for resected atypical and anaplastic meningiomas is typically delivered as fractionated RT, although SRS has been increasingly used either as adjuvant treatment or, more frequently, as salvage treatment for recurrent tumors [15–28, 81, 82, 84–86]. A summary of selected published series for atypical and anaplastic meningiomas is shown in Table 3.

**Table 3:** Summary of main published studies on stereotactic radiosurgery for the treatment of grade 2and grade 3 meningiomas

Authors	Patients (grade, no)	Tumor volume (cm3)	Median dose Gy	Median follow-up months	Progression-free survival, %	Overall survival, %	Toxicity
Kondziolka et al. 2008 [46]	29	NA	14	48	NA	22 at 5 years	7%
Choi et al. 2010 [14]	G2 (*n*=25)	5.3	22	28	100 and 73 at 2 and 3 years.	NA	Grade 3 hydrocephalus (*n*=1)
El-Khatib et al. 2011 [23]	G3 (*n*=7) G2 (*n*=9)	NA	14	60	57 and 43 at 5 and 10 years	NA	3.5%
Pollock et al. 2012 [72]	G2 (*n*=37) G3 (*n*=13)	14.6	15	38	45 at 5 years	27 at 5 years	Radiation-related complications, 26%
Attia et al. 2012 [6]	G2 (*n*=24)	NA	14	42.5	40 and 25 at 2 and 5 years .	67 and 52 at 2 and 5 years	Grade II or more (*n*=2)
Hanakita et al. 2013 [31]	G2 (*n*=22)	6	18	23.5	NA	68 at 5 years	Facial dysesthesia (*n*=1).
Ferraro et al. 2014 [25]	G2 (*n*= 31) G3 (*n*=4)	3,9	18	34.5	70.1 (G2) and 0 (G3) at 3 years	83.4 (G2) and 33.3 (G3) at 3 years	NA
Bulthuis et al. 2014 [10]	G2 (*n*=34)	3,5	13	41	83.4 and 64.4 at 2 and 5 years	NA	Transient neurological complications (*n*=2)
Valery et al. 2016 [85]	G2 (*n*=18)	2.5	15	36	36 and 23 at 2 and 3 years	NA	Radionecrosis (*n*=2)
Wang et al. 2016 [87]	G2 (*n*= 37) G3 (*n*=9)	11.7	13.1	32.6	30 and 20 at 2 and 5 years	88.3 (G2) and 66.7 (G3) at 5 years	Neurologic symptoms, 26%
Liu X et al. 2018 [51]	75	NA	13	70	59.3 at 5 years	89.8 at 5 years	NA
Zhang G et al. 2016 [88]	131	NA	15	23.6	24 at 5 years	36.0 at 5 years	NA
Helis et al. 2020 [34]	48	2.49	15	44	45.8 at 5 years	74.7 at 5 years	Grade 3 or more, 27%
Kowalchuk et al. 2021 [47]	G2 (*n*=233)	6,1	15	37.6	53.9 and 33,1 at 3 and 5 years	NA	Neurologic symptoms, 21%
Sheppard et al. 2021	G3 (*n*=233) G2 (*n*=38)	7.5	14.8	37.8	66.6 and 33.6 at 2 and 5 years	77 and 62.4 at 5 and 10 years	Neurologic symptoms, 6.3%

*NA*, not assessed

Kowalchuk et al. [22] have recently reported the results of a large retrospective multicentric study of 233 atypical meningiomas treated with SRS. For high-risk grade 2 meningiomas, as defined by the RTOG 0539 study, the 3-year progression-free survival was 53.9%, being similar to the rate of 58.8% reported in the RTOG study. Hanakita et al. [18] reported 2-year and 5-year recurrence rates of 61% and 84%, respectively, in 22 patients treated with salvage SRS. Analysis of prognostic factors showed that a tumor volume < 6 ml, margin doses > 18 Gy, and a Karnofsky Performance Status score of ≥ 90 were associated with a better outcome. Attia et al. [15] reported the clinical outcomes of 24 patients who received Gamma Knife SRS as either primary or salvage treatment for patients with atypical meningiomas using a median marginal dose of 14 Gy. With a median follow-up time of 42.5 months, local control rates at 2 and 5 years were 51% and 44%, respectively. Eight recurrences were in-field, four were marginal failures, and two were distant failures. In another retrospective series of 44 patients who received Gamma Knife SRS early after surgery or at tumor recurrence, Zhang et al. [28] showed 5-year actuarial local control and overall survival rates of 51% and 87%, respectively, at a median follow-up time of 51 months. Serious neurological complications occurred in 7.5% of patients. Similar results have been reported by others (Table 3).

A few studies have evaluated the efficacy of SRS for patients with anaplastic meningiomas [17, 21, 24, 25, 27]. In an international, multicenter, retrospective study of 271 patients with atypical (*n*=233) and anaplastic meningioma (*n*=38) treated with Gamma Knife SRS with a median dose of about 15 Gy, Shepard et al. [25] reported progression-free and overall survival rates of 33.6% and 77.0%, respectively, at 5 years. For patients with anaplastic meningiomas, increased age and reduced KPS (HR 0.95, *p* = 0.04) were associated with shorter OS. In another small series of 29 patients who received post-operative SRS with a mean margin dose of 14 Gy, Kondziolka et al. [21] reported progression-free survival rates of 17% at 15 months and 9% at 60 months. In contrast, El-Khatib et al. [17] reported higher rates of progression-free survival, 57% at 3 years and 43% at 10 years, for 7 patients with anaplastic meningiomas receiving Gamma Knife SRS with a margin dose of 14 Gy.

Hypofractionated SRT, typically 24–30 Gy given in 3 to 5 fractions, has also been employed as an alternative to single-fraction SRS for brain tumors, generally for larger or critically located tumors, e.g., involving the anterior optic apparatus, or the sagittal sinus [14]. Presently, hypofractionated SRT data specific to atypical meningioma is limited. The reported local control reported in few series has been essentially equivalent to single-fraction SRS, possibly with a lower risk of side effects [28, 89]. Vernimmen et al. [89] reported the outcome of stereotactic hypofractionated proton beam RT in 18 patients with skull base meningiomas. With a median follow-up of 31 months, 88% of tumors remained under control, even though large tumors up to 63 ml were treated.

Overall, data from the literature suggest that SRS is a feasible and safe treatment for patients with atypical and anaplastic meningiomas, especially for relatively small recurrent tumors less than 3 cm in size. Based of the scarcity of published data, its superiority over fractionated RT as well as its efficacy for patients with anaplastic meningiomas remains unsustained. Hypofractionated SRT may represent an alternative to single-fraction SRS for larger tumors or in the proximity of critical areas with the aim of limiting the potential treatment-related toxicity.

### Proton beam RT

Several studies have reported the outcome of proton beam and carbon ion therapy for atypical and anaplastic meningiomas [63, 64, 67, 80, 87, 90–92]. In a recent systematic review, Coggins et al. [51] reported the results of ion RT in maintaining local control in atypical and anaplastic meningiomas. With a mean follow-up time ranging from 60 to 145 months, mean local control rates following proton beam therapy were 59.6% at 5 years, accounting for a total of 82 patients included in 6 studies. Across the studies reporting on carbon ion RT, local control was 54% at 12 months and 33% at 24 months. A summary of studies reporting clinical outcomes of patients with atypical and anaplastic meningioma following proton and carbon ion RT is summarized in Table 4 [63, 64, 67, 80, 87, 90–92].

**Table 4:** Summary of main published studies on particle therapy for grade 2 and grade 3 meningiomas

Authors	Patients (grade, no)	Treatment modality	Median dose GyE	Median Follow-up (months)	Progression-free survival, %	Overall survival,%	Toxicity
Slater et al 2012 [79]	G2, 4 G1, 47	Proton	59	74	G2,50 at 5 years GI, 99 at 5 years	NA	6 patients developed neurologic symptoms
Adeberg et al 2012 [1]	G2, 62 G1, 23	FSRT, IMRT, Carbon Ion	57.6	73	G2, 95 and 50, G3, 63 and 13, at 2 and 5 years	81 (G2) and 53 (G3) at 5 years	NA
Combs et al*.* 2013 [18]	G2/3, 36	Photon RT and a carbon ion boost	Photon RT, 50; carbon ion boost, 18	12	54 and 33 at 1 and 2 years	NA	No grade 3/4 toxicity
Combs et al*.* 2013 [19]	G2, 23 G3, 4	Carbon ion	Photon RT 50; carbon ion boost, 18	6	81 at 12 months	NA	No grade 3/4 toxicity
Murray et al*.* 2017 [61]	G2, 33 G3, 2	Proton	62.0	56.9	68 at 5 years	80.7 at 5 years	Optic tract and pituitary toxicities, fatigue impaired hearing
El Shafie et al*.* 2018 [24]	G2, 25 G3, 6	Proton and carbon ion boost	Photon, 54; carbon ion boost, 18	49.7	71 and 56.5 at 1 and 2 years	89.6 at 1year 71.4 at 2 years	No grade IV or V toxicities

*RT*, radiation therapy; *IMRT*, intensity-modulated radiation therapy; *FSRT*, fractionated stereotactic radiotherapy; *NA*, not assessed

With regard to the limited number of studies and patients, proton and carbon ion therapy maintain comparable rates of local control to conventional photon therapy. Prospective trials remain necessary to quantify the efficacy of ion beam RT versus conventional photon therapy in terms of local control, overall survival, and treatment-related toxicity rates. The NCT01166321 phase II open-label trial is currently recruiting patients with atypical meningiomas undergoing partial resection (Simpson 4 and 5) treated with carbon ion boost in combination with photon RT. Other clinical trials have been recently activated or are currently recruiting patients in order to test the efficacy of carbon ion therapy in atypical meningioma (NCT01166321) and proton dose escalation in atypical and anaplastic meningiomas (NCT02978677).

### Radiation dose and timing of RT

Radiation dose and timing of RT represent important variables for the clinical outcome of atypical meningiomas. Conventionally fractionated RT with total doses of 54–60 Gy given in 1.8–2.0 Gy fractions is usually utilized in the majority of published series. A few studies employing doses ≥ 60 Gy showed improved local control [10, 35, 57, 89], whereas doses of 54–57 Gy [42, 59] or less than 54 Gy [2, 42, 52] were apparently associated with low or no benefits. As with atypical meningioma, higher RT doses appear to improve local tumor control for patients with anaplastic histology [69, 74]. For patients receiving SRS, single doses of 14–18 Gy are typically used in the majority of radiation centers with similar local control (Table 3); in contrast, doses ≤ 12 Gy have been associated with inferior local control rates [77]. Kano et al. [19] used SRS as salvage therapy for recurrent tumors after surgical failure and showed a dose-dependent improvement of 5-year progression-free survival for patients with both atypical and anaplastic meningiomas. Survival rates increased from 29.4 to 63.1% when recurrent tumors received a marginal radiation dose exceeding 20 Gy compared to 15 Gy. Differently from single-fraction SRS, no studies have evaluated the impact of different hypofractionated schedules for grade 2 or 3 meningiomas.

In summary, higher doses given in conventional fractionation seem to provide better overall outcomes compared with lower doses; however, no controlled prospective studies have directly compared different doses and significant survival advantages observed with higher doses remain to be confirmed in controlled studies. Similarly, RT modalities have not been compared in well-designed studies to provide evidence of the superiority of one treatment modality over the others.

With regard to the timing of RT for atypical meningiomas, postoperative RT seems more effective when administered adjuvantly rather than at recurrence, and most authors recommend this approach [35–37, 42, 47, 48, 52, 60, 89]. In the study of Lee et al. [22], adjuvant RT was associated with a longer time of tumor progression compared with salvage RT. For patients with unresectable and symptomatic meningioma or with imminent risk of symptomatology in case of further progression, there is a general consensus that RT should be initiated as soon as possible [6]. Interestingly, Islim et al. [93] developed a prognostic model to guide personalized monitoring of incidental asymptomatic meningioma patients. By combining data on patient characteristics (age, performance status, and co-morbidities) and MRI features, including tumor hyperintensity**,** peritumoral edema, proximity to neurovascular structures, and size, they proposed an individualized monitoring strategy for patients with low, medium, or high risk for tumor progression, developing a calculator which is freely available (https://www.impact-meningioma.com).

After gross total resection, the 5-year and 10-year progression-free survival rates were 94% for both in the adjuvant RT group versus 42% and 36%, respectively, in the salvage RT group. Results of ROAM/EORTC 1308 trial which are expected in 2025 will help to better define the postoperative management of these patients.

### Reirradiation

Thanks to the continuous improvement in radiation science and technology, reirradiation has emerged as a feasible approach for patients with different brain tumors [55]. Few retrospective studies have reported the feasibility of reirradiation for patients with recurrent meningiomas [66, 68, 88]. In a series of 43 patients receiving a second course of RT, Lin et al. [74] showed local control, progression-free survival, and overall survival rates of 77%, 60%, and 87% at 1 year, and 70%, 43%, and 68% at 2 years, respectively, for grade 2 and grade 3 meningiomas, with no significant differences between fractionated RT and SRS. The treatment was associated with an acceptable toxicity profile, with 15% of patients who developed grades 2 to 4 radionecrosis. This is consistent with previous studies on reirradiation of brain gliomas suggesting that the risk of symptomatic brain necrosis is low if the cumulative equivalent dose of 2 Gy per fraction (EQD2) is less than 100 Gy [88].

Overall, a few studies support the role of reirradiation as a feasible treatment option for selected patients with recurrent atypical and anaplastic meningiomas that recurred after previous standard treatment. Prospective studies with appropriate follow-up are needed to validate the favorable impact of reirradiation, delivered either as fractionated SRT or as SRS, for recurrent meningiomas.

### Toxicity

The reported toxicity after postoperative RT for atypical and anaplastic meningiomas is modest; using typical doses of 54–60 Gy, toxicity ranges from 0 to 17% and includes radiation-induced brain necrosis (0–15%), visual disturbances (2–5%), hypopituitarism (5–30%), and cognitive disturbance (2–17%) (Tables 1, 2, 3, and 4). In the EORTC 22042–26042 observation study, the rate of the late adverse effect of the Common Terminology Criteria for Adverse Events grade 3 or more associated with adjuvant RT following gross total resection for atypical meningioma was 14.3% with no toxic death using a radiation dose of 60 Gy is given in 2 Gy per fraction [57]. In the NRG Oncology/RTOG0539 trial reporting the clinical outcome for 53 patients who received IMRT with a dose of 60 Gy given in 30 fractions for a high-risk meningioma, Rogers et al. [10] reported combined acute and late adverse events in about 40% of patients, although they were limited to grades 1 to 3, except for a single necrosis-related grade 5 event at a median follow-up of 4 years. Of note, only grade 1 and 2 adverse events occurred in patients with intermediate-risk meningiomas who were treated with IMRT or 3D conformal RT using doses of 54 Gy given in 30 fractions in the same trial [4]. A similar acceptable incidence of radiation-related toxicity has been reported in the majority of published studies of conventionally fractionated RT including either atypical or anaplastic meningiomas (Tables 1 and 2). For patients receiving SRS, neurological toxicity rates up to 26% have been reported in few studies [22, 24, 27, 82], although it remains below 10% when limited volumes are treated [15, 17, 21]. Potential neurocognitive toxicity of adjuvant RT is a major cause that makes physicians hesitate to apply it to patients with an atypical meningioma after gross total resection. The incidence of neurotoxicity ranges from 3.4 to 16.7% according to the location of the lesion, radiation dose, and radiation modality, although no published studies have evaluated neurocognitive changes after RT using formal neuropsychological testing.

In general, studies support the safety of radiation treatment given adjuvantly or at recurrence. The impact of advanced techniques for RT such as IMRT and VMAT can lead to improvement in safety profile. Conventionally fractionated RT is usually employed as adjuvant treatment for patients with large resection cavity or large recurrent tumors, while SRS or hypofractionated schedules may represent a feasible treatment option for small-to-moderate tumors, usually less than 3 cm or not in close proximity to sensitive brain structures, such as brainstem or optic apparatus.

## Conclusions

At present, surgery retains a central role in the management of atypical and anaplastic meningiomas. For most patients, gross total resection remains the benchmark, although total surgical excision within the constraints of acceptable morbidity is not always achievable. Postoperative RT is usually recommended after subtotal resection, with several studies indicating improvements in local control up to 70% at 5 years. Similar rates have been shown after SRS; however, the latest is usually offered to patients with smaller-to-moderate recurrent tumors. Controversy exists regarding the role and the efficacy of postoperative adjuvant RT in patients receiving gross total resection. The relatively divergent results in the literature are most likely explained by the retrospective nature of the series and the relatively small number of patients evaluated. Keeping this in mind, EORTC 22042-26042 and RTOG 0539 prospective trials have already confirmed an excellent patients’ outcome, with approximately 90% progression-free survival rates at 3 years for WHO grade 2 meningioma undergoing complete resection and adjuvant high-dose RT, depending on patient- and tumor-treatment-related factors. Additional studies should better elucidate the timing, the optimal dose/fractionation, and radiation technique for these tumors. The development of a molecularly based classification of meningiomas will provide a better understanding of tumor biology and could help us predict which patients will benefit from adjuvant therapy.
